# Multiple microvenular hemangioma: A case report

**DOI:** 10.3892/ol.2013.1659

**Published:** 2013-11-05

**Authors:** DONG FANG AI, YAN LI, AIKAJ JINDAL, PING LI

**Affiliations:** 1 Department of Dermatology, Cangzhou Central Hospital, Cangzhou, Hebei 061001, P.R. China; 2 Department of Dermatology, Tianjin Medical University, General Hospital, Tianjin 300052, P.R. China; 3 Tianjin Medical University, Tianjin 300052, P.R. China

**Keywords:** microvenular hemangioma

## Abstract

The current study reports a case of multiple microvenular hemangioma (MH). A 35-year-old male presented with dark red maculopapules on the trunk and limbs that had been apparent for 5 years. The number of lesions exceeded 100 in total. A histological examination demonstrated multiple, irregular, branching venules in the dermis, without any endothelial atypia. On immunohistochemical analysis of the vascular structures, the endothelial cells stained positive for CD31, CD34 and factor VIII, and the perivascular cells stained positive for SMA and HHF-35. These observations were consistent with a diagnosis of MH, and should be differentiated from the most common differential diagnosis of patch-stage Kaposi’s sarcoma. There was no clear effect following topical application of recombinant human interferon α-2b gel.

## Introduction

Microvenular hemangioma (MH) is an acquired benign vascular tumor, occurring on the trunk and limbs in young to middle-aged adults without any gender predilection. MH usually presents as a solitary, purple-to-red papule/plaque measuring 5–20 mm in diameter. MH was first described by Bantel *et al* in 1989 (1). The etiology of MH is unknown. MH is rare, with <50 cases reported to date. Even in these reported cases, accounts of multiple MH are extremely uncommon. The present study reports a case of multiple MH. Informed consent was obtained from the patient.

## Case report

### Patient presentation

**Figure 1:**
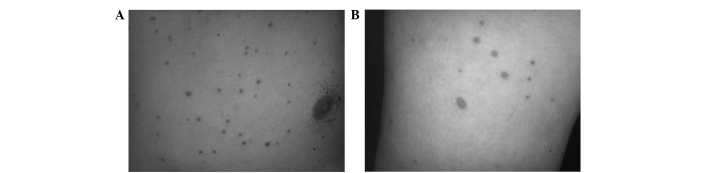
(A) Dark red maculopapules on the chest. (B) Multiple dark-red maculopapules on the lower extremities.

A 35-year-old male presented to Cangzhou Central Hospital (Cangzhou, China) in September 2009 with multiple dark red maculopapules on the trunk and limbs (Fig. 1). The patient reported a history of their presence on the trunk and limbs for the past 5 years and with the absence of any precipitating factor. Initially, the lesions were the size of rice granules, painless and itchy, although not troublesome enough to persuade the patient to attend a consultation. Thereafter, the size of the lesions gradually enlarged and also increased in number. Subsequently, the individual sought medical advice at a local hospital and was prescribed oral antihistamines and topical corticosteroids that proved of no benefit. No other family member demonstrated such lesions.

### Lesion examination

Upon examination, numerous dark red, circular, non-scaly maculopapules measuring 5 mm in diameter were noted on the chest, back, abdomen, buttocks and upper and lower extremities. The lesions had a diffuse distribution with clear borders and were painless. An attempt to count the number of lesions found >100. The lesions were found in a greater concentration on the chest and back. The head, face, palms and soles were unaffected.

### Clinical and pathological analyses

Nothing abnormal was found on chest X-ray or abdominal B-mode ultrasound scan. The patient’s blood profile, urine and stool routine, liver function, renal function and electrolyte levels were also normal.

### Patient diagnosis

**Figure 2:**
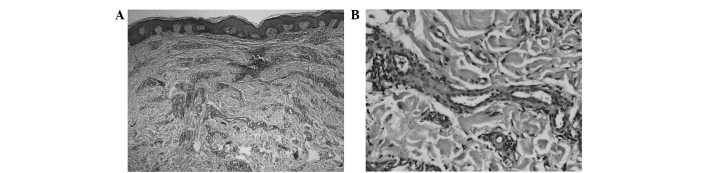
Hematoxylin-eosin stain of small, irregularly branched blood vessels proliferating throughout the dermis and embedded in a desmoplastic stroma at magnifications (A) ×40 and (B) ×200.

Two lesions of the chest were removed completely for diagnosis. The tumors were of the same histopathology and demonstrated proliferation of irregularly branched, thin-walled venules infiltrating the sclerotic dermal collagen and the presence of few blood vessels with inconspicuous lumina (Fig. 2A). A mixture of flat and plump endothelial cells was observed (Fig. 2B), however, there was an absence of cellular atypia and mitotic figures. Perivascular infiltration of few lymphocytes was noted.

**Figure 3:**
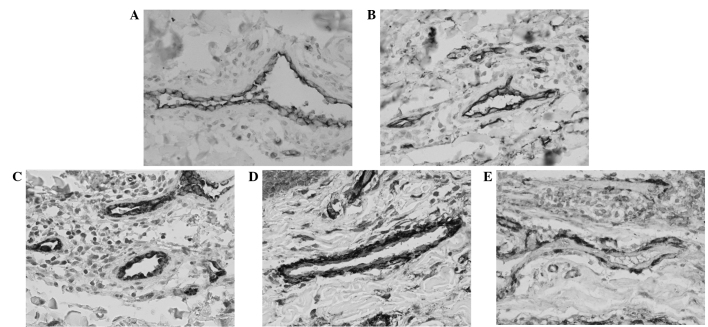
Endothelial cells of the proliferative vessels expressed (A) CD31, (B) CD34 and (C) factor VIII. (D) The pericytes expressed SMA and (E) HHF-35 (magnification, ×400).

Immunohistochemically, the endothelial cells of the proliferating vessels expressed CD31 (Fig. 3A), CD34 (Fig. 3B) and factor VIII (Fig. 3C). The pericytes expressed SMA (Fig. 3D) and HHF-35 (Fig. 3E). The diagnosis was microvenular hemangioma. The patient was treated with recombinant human interferon α-2b gel, twice a day. There was no clear effect after a month.

## Discussion

MH is an acquired benign vascular tumor. MH was first described by Bantel *et al* in 1989. At that time, the study reported microcapillary angiomas in three female patients (1). In 1991, similar cases were described by Hunt *et al* using current terminology (2). MH is rare, with ≤50 cases reported to date.

The etiology of MH is unknown, but in specific cases, MH has been observed following changes in hormonal contraception or during pregnancy (1). Sex hormone imbalance may also account for certain cases (3). MH shows a predilection for young to middle-aged adults, but is also identified in children (4). It is most frequently located on the trunk and upper extremities, and less frequently on the toes (5). MH grows slowly and usually presents as a solitary, purple to red papule/plaque measuring 5–20 mm in diameter. Alternatively, it can also present as a pale red nodule, which clinically resembles cutaneous inflammation (6). Generally, the lesion is asymptomatic, but slight tenderness has also been noted in specific cases (7). Multiple MHs, as in the present case, are extremely rare. To date, Xu *et al* has already reported four cases of patients from China who exhibited a rapidly progressive abrupt onset of numerous MHs numbering in the tens to hundreds (8).

Upon dermoscopic examination, multiple well-demarcated red globules are observed with the presence of a fine pigmented network at the periphery (9). Histologically, MH is composed of thin-walled, irregularly branching venules with inconspicuous vascular lumina. The collagen bundles in the dermis are thickened. The endothelial cells are surrounded by pericytes (10) and may present as a mixture of flat or plump cells, but with a lack of cellular atypia, pleomorphism or mitotic figures (11). Immunohistochemically, the endothelial cells of an MH are positive for CD31, CD34 and factor VIII, and the pericytes are positive for SMA (9,12), but both stain negative for podoplanin (13).

The tumor may exhibit infiltrative growth throughout the dermis. Therefore, pathologists and clinicians must be aware of the existence of this type of infiltrative growth accompanying the hemangioma and hence, avoid overdiagnosis and overtreatment (11).

There have been multiple accounts of MH associated with other systemic diseases in the medical literature. An association with a case of POEMS syndrome was previously reported by Hudnall *et al*(14) where HHV-8 was directly demonstrated within the endothelial cells of MH. It is worth noting that the hemangiomas in this case responded to chemotherapy with cyclophosphamide and prednisone.

A previously reported case of MH in a young child with acute myelogenous leukemia (4) demonstrated an association with systemic immunosuppression. In addition, Rikihisa *et al* reported a case of MH in a patient with Wiskott-Aldrich syndrome (15).

Histologically, the most common differential diagnosis of MH is the patch stage of Kaposi’s sarcoma (KS) (2). However, in KS, irregular vascular spaces undergo anastomosis rather than collapse, as seen in MH. The presence of accompanying atypical endothelial cells, eosinophilic hyaline globules, plasma cells and fascicles of spindle cells favors the diagnosis of KS over MH (9).

Additional differential diagnoses include multinucleate cell angiohistiocytoma (MCA) and reactive angioendotheliomatosis (RAE). In MCA, the characteristic cells are large, with irregular, unusually-shaped, scalloped or angular margins (16). The RAE is characterized histologically by the proliferation of endothelial cells within the vascular lumina, resulting in the obliteration of the involved vessels secondary to intravascular thrombi, thereby, making it easier to determine the diagnosis. In addition, MH must be distinguished from other entities, including targetoid hemosiderotic hemangioma, tufted angioma, sclerosing angioma and granuloma pyogenicum (9,10,12).

The distinctive feature of the present case that makes it an important academic observation is that, besides the multiple skin lesions, there are also epidermal changes on the histopathology, which include spreading and interdigitating epidermal processes and an increase in the basal lamina pigmentation. Furthermore, the course of this case was longer than usually observed in MH.

For cases of this disease with minor solitary lesions, excision has been associated with cure and usually no recurrence is observed. In cases with major or multiple skin eruptions, regular follow-ups should be performed, although the disease is self-limiting in nature. In the present case, some primary lesions disappeared without further treatment; however, a few new eruptions appeared on the patient’s back and chest.
